# Global trends and hotspots in exercise therapy for insomnia research: bibliometric and visual analysis

**DOI:** 10.3389/fneur.2025.1412152

**Published:** 2025-04-23

**Authors:** Liang Li, Jiuzhu Liang, Tonggang Fan

**Affiliations:** College of Wushu, Shanghai University of Sport, Shanghai, China

**Keywords:** exercise, insomnia, sleep, quality of life, bibliometrics

## Abstract

**Objective:**

This study analyzes trends and hotspots in the research on exercise for insomnia using bibliometric methods and visually presents key information in the field.

**Methods:**

Using Web of Science Core Collection as a source of literature, Microsoft Excel 2019, CiteSpace, VOSviewer, and the Bibliometrix package based on R language software were used to create visualization graphs and analyze the publications by country and region, institution, journal, author, reference, and keyword.

**Results:**

This study included 1,419 papers. The overall number of publications showed an increasing trend, and the highest number of papers in this field were published by the United States and China by country, the University of California System in the United States by institution, and Markus Gerber from Switzerland by author. The trends and hotspots in this field identified through keyword analysis include “insomnia,” exercise,” “depression,” “older adults,” and “quality of life.”

**Conclusion:**

The research field of exercise therapy for insomnia is receiving increasing attention, and this study provides a clear and intuitive reference for researchers.

## 1 Introduction

Insomnia refers to the inability to fall or stay asleep, resulting in sleep deprivation. It a common sleep disorder (1, 2) and health problem globally (3). Surveys show that approximately two billion people worldwide have sleep problems, with 35% of Americans suffering from insomnia (4). It is important to note that the incidence of insomnia and sleep problems is increasing annually, which is related to various factors in the modern society, such as the pace of life, work, and study stress (5, 6). With the continuing changes in social environment and lifestyle, insomnia has become one of the key issues addressed in public health.

Currently, insomnia is mainly treated pharmacologically and non-pharmacologically. Taking medication for a long period can jeopardize patient health, affect mood swings, create dependency on medication, and cause other side effects (7). As a non-pharmacological complement and alternative to insomnia treatment, exercise has a demonstrated a positive impact on patients (8, 9). Numerous clinical studies have shown that exercise can improve insomnia symptoms, immunity, and sleep quality (10–12). Exercise as an adjunct to insomnia treatment has received increasing attention and in-depth research (13). However, even though a relatively large number of studies support the effectiveness of exercise therapy in the management of insomnia, there is still a great deal of controversy in the existing literature regarding the specific intervention modalities, criteria for evaluating the effectiveness of exercise therapy, and most effective types of exercise. As the amount of relevant literature continues to increase with research on insomnia and exercise therapy, however, how to systematically summarize these findings and identify trends and hotspots in global research remains an urgent issue. Therefore, it is necessary to analyze and study developmental trends and research hotspots in the field of exercise for insomnia to provide a systematic and comprehensive reference for researchers.

Bibliometric analysis is a research method that uses statistical techniques to describe and evaluate information in the literature quantitatively. This method quantifies the literature’s external characteristics and internal contents to reveal the quantitative distribution, development trend, internal connection, and law, thus providing a quantitative basis and support for academic research and knowledge management (14, 15). Therefore, this study aims to analyze the global research trends and hotspots in exercise therapy for insomnia through bibliometric and visual analyses, identify the main directions of current research, and explore potential research gaps in the future, with a view to providing valuable references for scholars and clinicians in related fields.

## 2 Methods

### 2.1 Data collection

The Web of Science Core Collection (WOSCC) is widely recognized as one of the world’s most authoritative and reliable comprehensive academic literature databases; it summarizes taxonomic journals issued by renowned publishers and institutions around the world. We used WOSCC as the data source for this study. To ensure that we retrieve comprehensive literature in the research field of exercise therapy for insomnia, we first searched for terms similar to “exercise therapy” and “insomnia” in the Medical Subject Headings of the PubMed Search Information Supplement and then developed a refined search.

The search was conducted on March 18, 2024. The search period was from January 1, 2010, to December 31, 2023; the literature types were limited to articles or reviews, and the language was limited to English. Regarding the number of documents, we retrieved (1) 220,676 documents by searching for expressions related to “exercise” and (2) 27,652 documents by searching for expressions related to “insomnia”; moreover, the retrieved documents related to “exercise” and “insomnia” were searched again using AND concatenation, and (3) 1,419 documents were retrieved. Finally, these 1,419 documents were used to analyze the research field of exercise therapy for insomnia. Table 1 describes the specific search method.

**Table 1:** Data retrieval methods.

Content		
Data sources	WoSCC	
Time span	2010 ~ 2023	
Languages	English	
Literature types	Article or Review	
Search strategy	#1(220676)	[TS = (“Exercises”OR“Physical Activity”OR “Activities, Physical”OR“Activity, Physical”OR “Physical Activities”OR“Exercise, Physical”OR“Exercises, Physical”OR“Physical Exercise”OR“Physical Exercises”OR“Acute Exercise”OR“Acute Exercises”OR“Exercise, Acute”OR“Exercises, Acute”OR“Exercise, Isometric”OR“Exercises, Isometric”OR“Isometric Exercises”OR“Isometric Exercise”OR“Exercise, Aerobic”OR“Aerobic Exercise”OR“Aerobic Exercises”OR“Exercises, Aerobic”OR“Exercise Training”OR“Exercise Trainings”OR“Training, Exercise”OR“Trainings, Exercise”OR“qigong”OR “Qi Gong”OR“Ch’i Kung”OR“taiji”OR“Tai-ji”OR“Tai Chi”OR“yoga”)]
	#2(27652)	[TS = (“Primary Insomnia” OR “Insomnia, Primary” OR “Transient Insomnia” OR“Insomnia, Transient” OR “Secondary Insomnia” OR“Insomnia, Secondary” OR“Sleep Initiation” OR “Sleeplessness” OR “Insomnia Disorder” OR “Insomnia Disorders” OR “Insomnia”OR “Insomnias” OR “Chronic Insomnia” OR “Insomnia, Chronic”)]
	#3(1419)	#1 AND #2

### 2.2 Data analysis

The research area of exercise therapy for insomnia was analyzed using Microsoft Excel 2019, CiteSpace, VOSviewer, and the Bibliometrix package based on R language. Microsoft Excel 2019 was used to produce publication trend histograms for yearly publication trends. Analyses of countries/regions and keywords were used to create visualization graphs using the Bibliometrix package in R language. In the keyword analysis, VOSviewer was used to visualize keyword density. Moreover, CiteSpace was used to produce the institution visualization, author co-citation visualization, and reference clustering graphs.

Co-occurrence analysis was conducted to reveal the correlation and structural relationships between institutions, authors, and keywords in the field of exercise for insomnia by analyzing the frequency of their co-occurrence in the same or different documents. Co-occurrence analysis clearly revealed research hotspots, development trends, and cross-fertilization between different keywords in this field. Moreover, cluster analysis was conducted to reveal the inherent structure and patterns of a field of study by grouping similar objects to form different research themes or directions.

## 3 Results

### 3.1 Publication

**Figure 1:**
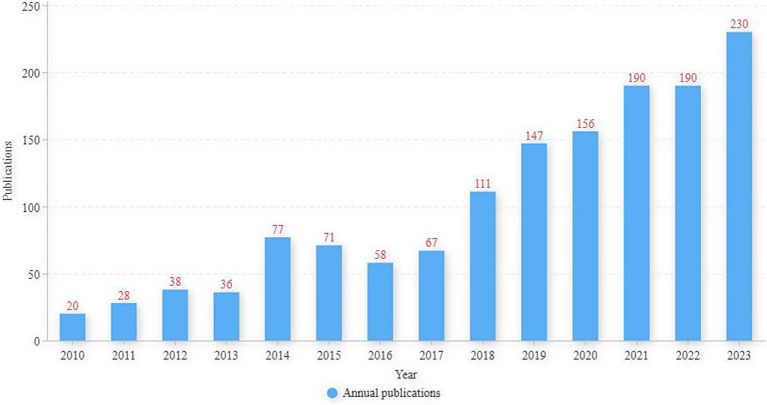
Annual publications in the field of exercise therapy for insomnia research.

Of the final 1,419 publications included in this study, 1,170 were articles and 249 were reviews. Figure 1 shows the annual number of publications in the field of exercise therapy for insomnia from 2010 to 2023. Although the number of publications fluctuated, the overall number showed a growing trend, from 20 published articles in 2010 to 230 in 2023, representing an 11-fold growth rate. The number of publications increased per year since 2018, indicating that the number of people involved in exercise therapy for insomnia research is growing, and researchers are increasingly emphasizing this field of study.

### 3.2 Countries and regions

**Figure 2:**
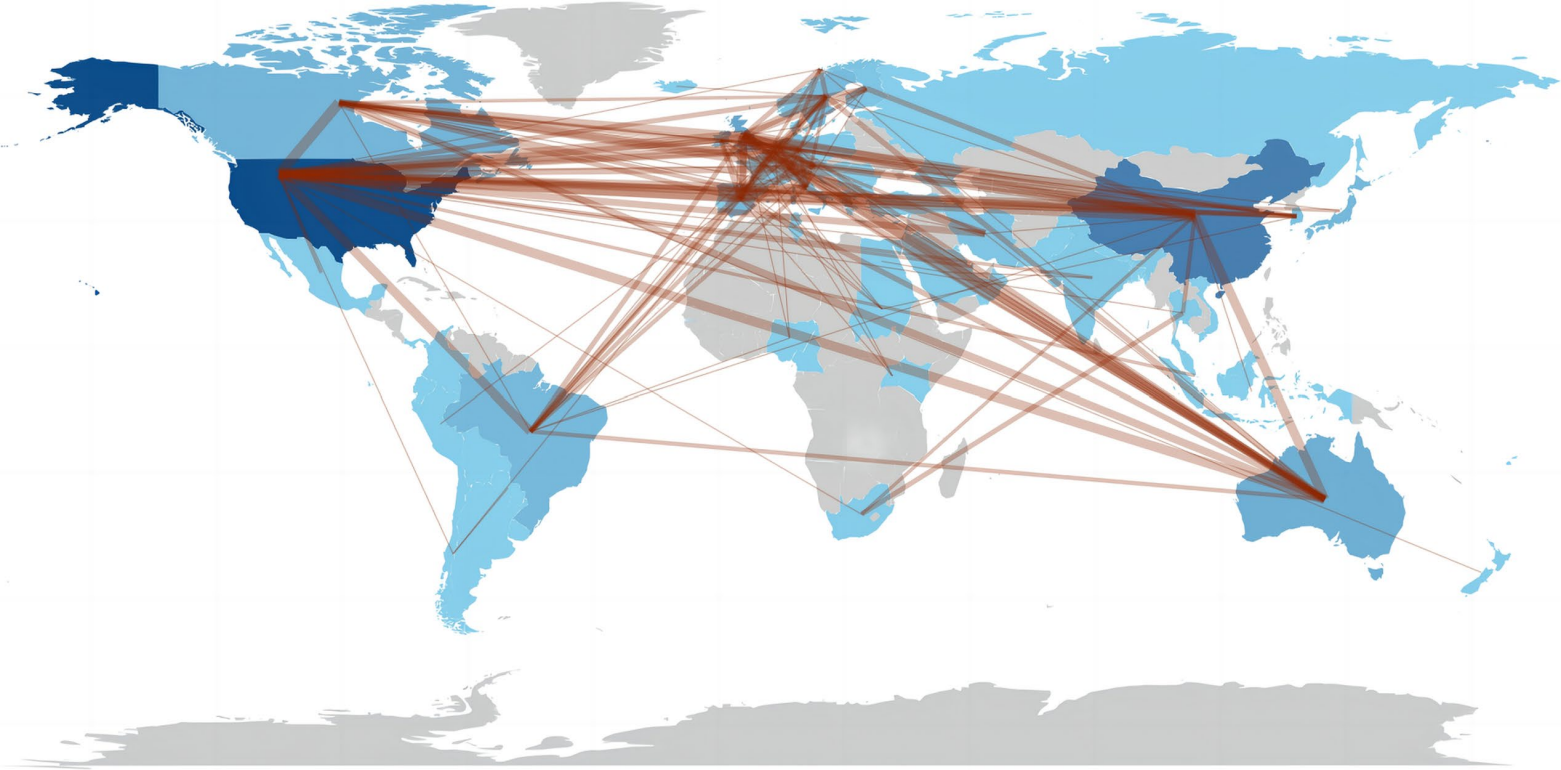
Distribution of publications by country and region.

Figure 2 illustrates the distribution of 422 countries/regions across six continents conducting research on exercise therapy for insomnia. The publications are concentrated in Western Europe, East Asia, and North America. Table 2 shows that the country with the highest number of publications is the United States (423 articles), followed by China (213 articles). In contrast, the number of publications in other countries is much lower. Moreover, Table 2 shows that the United States and China have the closest connections in this field, and the United States cooperates closely with China, the United Kingdom, Canada, and Australia. Therefore, China, based on a comparison with United States, should strengthen and establish cooperative relationships with other countries in this research field.

**Table 2:** Number of publications and frequency of collaboration by country and region.

Rank	Region	Frequency	Rank	From	To	Frequency
1	USA	423	1	USA	China	38
2	China	213	2	China	United Kingdom	27
3	Australia	127	3	USA	United Kingdom	23
4	UK	101	4	USA	Canada	22
5	Japan	80	5	United kingdom	Spain	18
6	Canada	72	6	USA	Australia	18
7	Spain	68	7	Australia	United Kingdom	16
8	Brazil	68	8	China	Australia	16
9	Sweden	62	9	USA	Brazil	16
10	Italy	60	10	China	Canada	12

### 3.3 Institutions

Table 3 shows the top 10 institutions with the highest number of contributions in the research field of exercise for insomnia, with the University of California System (68 articles), an institution from the United States, ranking first. This institution is followed by Harvard University (48 articles) and Pennsylvania Commonwealth System of Higher Education (42 articles). Of the top 10 institutions, six are from the United States, two are from China, one is from Sweden, and one is from the United Kingdom. This indicates that an increasing number of institutions in the United States engage in research on exercise therapy for insomnia.

**Table 3:** Top 10 organizations with the highest number of publications.

Rank	Institutions	Counts	Country	Centrality
1	University of California System	68	USA	0.34
2	Harvard University	48	USA	0.17
3	Pennsylvania Commonwealth System of Higher Education	42	USA	0.23
4	US Department of Veterans Affairs	30	USA	0.11
5	Harvard Medical School	28	USA	0.23
6	University of Basel	28	Swit	0.11
7	University of London	27	UK	0.08
8	Veterans Health Administration	26	USA	0.01
9	Hong Kong Polytechnic University	22	China	0.08
10	Univerdity of Hong Kong	22	China	0.00

**Figure 3:**
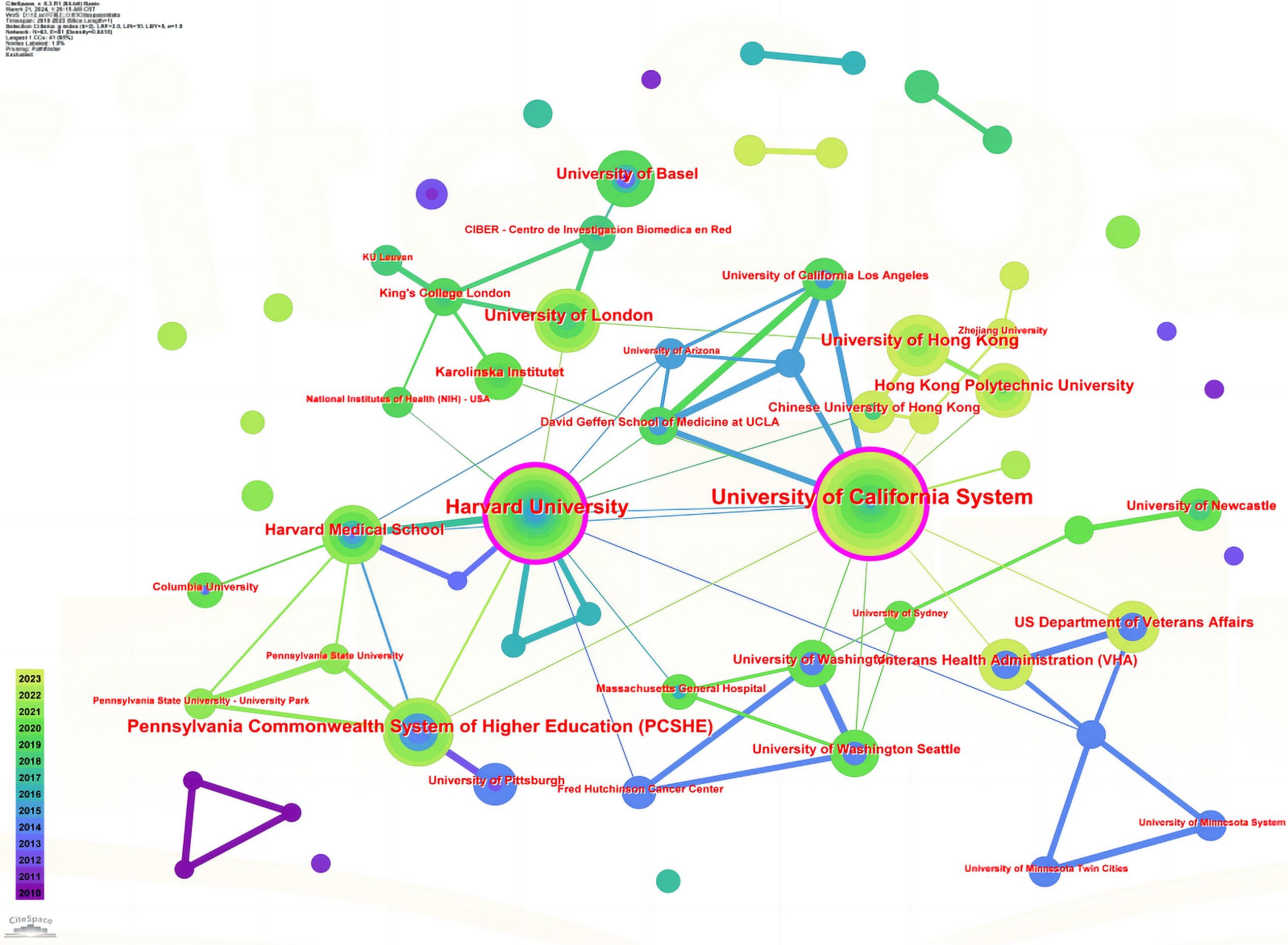
Visualization of institutional cooperation.

Figure 3 shows the visualization of institutional collaboration in research on exercise therapy for insomnia. The University of California System has a strong collaborative network and relationships with other institutions.

### 3.4 Journals

A total of 1,419 articles on exercise therapy for insomnia have been published in 563 journals during the target period. Table 4 shows the top 10 journals with the highest number of articles. The *International Journal of Environmental Research and Public Health* ranks first by publishing 50 studies related to exercise and insomnia, followed by *Sleep Medicine* (47 articles) and *PLOS One* (32 articles). The *Sleep Medicine Reviews* journal has the highest impact factor (10.5 for 2022) among these top 10 journals.

**Table 4:** Top 10 journals with the highest number of publications.

Rank	Journals	Counts	IF (2022)
1	INTERNATIONAL JOURNAL OF ENVIRONMENTAL RESEARCH AND PUBLIC HEALTH	50	4.6
2	SLEEP MEDICINE	47	4.8
3	PLOS ONE	32	3.7
4	JOURNAL OF SLEEP RESEARCH	28	4.4
5	SLEEP	27	5.6
6	SLEEP MEDICINE REVIEWS	27	10.5
7	BMJ OPEN	26	2.9
8	BEHAVIORAL SLEEP MEDICINE	20	3.1
9	FRONTIERS IN PSYCHIATRY	19	4.7
10	JOURNAL OF AFFECTIVE DISORDERS	18	6.6

### 3.5 Authors

A total of 7,768 authors contributed to research on exercise therapy for insomnia. Table 5 shows the top 10 authors with the highest number of publications in this field. This list is led by Markus Gerber (30 articles), followed by Serge Brand (22 articles) and Edith Holsboer-Trachsler (16 articles), all from the University of Basel. Four of the top 10 authors are from Switzerland, and three are from the United States, indicating that Switzerland and the United States have more outstanding people focusing on the research field of exercise therapy for insomnia.

**Table 5:** Top 10 authors with the highest number of publications.

Rank	Authors	Counts	Institution	Country	Centrality
1	Gerber, Markus	30	University of Basel	Switzerland	0.01
2	Brand, Serge	22	University of Basel	Switzerland	0.00
3	Holsboer-trachsler, Edith	16	University of Basel	Switzerland	0.00
4	Irwin, Michael R	14	University of California	USA	0.00
5	Duncan, Mitch J	11	Hunter Medical Research Institute	Australia	0.00
6	Puehse, Uwe	10	University of Basel Faculty of Medicine	Switzerland	0.00
7	Ensrud, Kristine E	8	University of Minnesota Twin Cities	USA	0.01
8	Plotnikoff, Ronald C	7	University of Newcastle	Australia	0.00
9	Tufik, Sergio	7	Universidade Federal de São Paulo	Brazil	0.00
10	Lacroix, Andrea Z	7	University of California	USA	0.00

**Figure 4:**
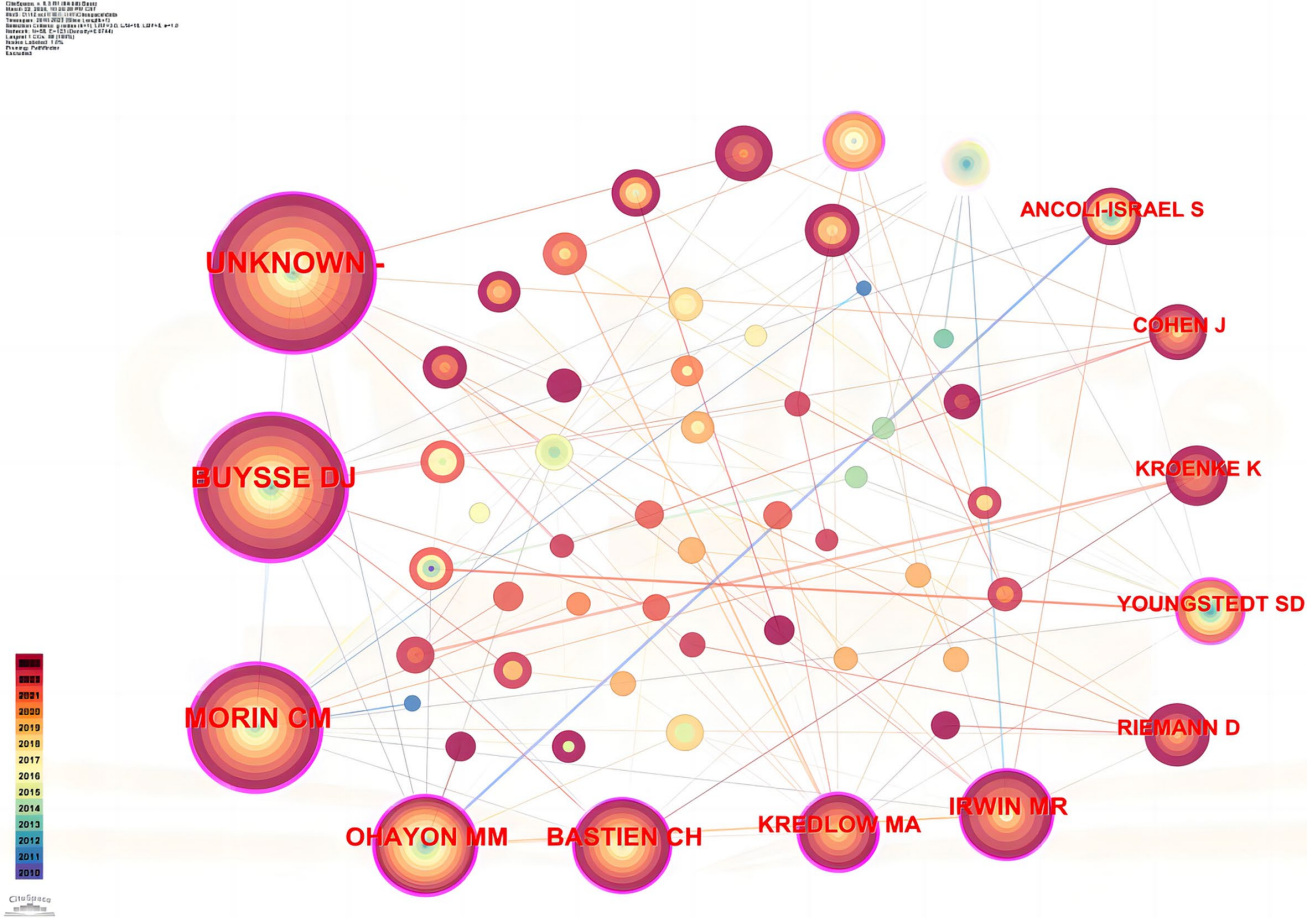
Author co-citation visualization.

Figure 4 shows the co-citation graph of authors who conducted research on exercise therapy for insomnia; the most cited author is Unknown, with 525 citations, followed by D. J. Buysse from the United States with 444 citations and C. M. Morin from Canada with 342 citations.

### References

3.6

Table 6 shows the top 10 most frequently cited references in the research field of exercise therapy for insomnia, of which seven are reviews and three are randomized controlled trials. The highest-ranked reference was Kredlow MA et al. (16) meta-analysis, which analyzed the effects of acute and regular exercise on sleep; this article was cited 62 times. This was followed by a review by Chennaoui M et al. (17) published in the *Sleep Medicine Review* journal; this article described the reciprocal fundamental physiological effects of sleep and exercises to improve the relevant applications of exercise in sleep medicine; this article was cited 38 times.

**Table 6:** Top 10 most-frequently cited references in exercise therapy.

Rank	Title	Author/year	Frequency	DOI
1	The effects of physical activity on sleep: a meta-analytic review	Kredlow MA/2015	62	10.1007/s10865-015-9617-6
2	Sleep and exercise: a reciprocal issue?	Chennaoui M/2015	38	10.1016/j.smrv.2014.06.008
3	Exercise can improve sleep quality: a systematic review and meta-analysis	Banno M/2018	35	10.7717/peerj.5172
4	European guideline for the diagnosis and treatment of insomnia	Riemann D/2017	33	10.1111/jsr.12594
5	Management of Chronic Insomnia Disorder in Adults: A Clinical Practice Guideline From the American College of Physicians	Qaseem A/2016	30	10.7326/M15-2175
6	The effect of resistance exercise on sleep: A systematic review of randomized controlled trials	Kovacevic A/2018	27	10.1016/j.smrv.2017.07.002
7	Aerobic exercise improves self-reported sleep and quality of life in older adults with insomnia	Reid KJ/2010	26	10.1016/j.sleep.2010.04.014
8	Cognitive behavioral therapy vs. Tai Chi for late life insomnia and inflammatory risk: a randomized controlled comparative efficacy trial	Irwin MR/2014	23	10.5665/sleep.4008
9	Sleep Disturbance, Sleep Duration, and Inflammation: A Systematic Review and Meta-Analysis of Cohort Studies and Experimental Sleep Deprivation	Irwin MR/2016	23	10.1016/j.biopsych.2015.05.014
10	Increased physical activity improves sleep and mood outcomes in inactive people with insomnia: a randomized controlled trial	Hartescu I/2015	22	10.1111/jsr.12297

**Figure 5:**
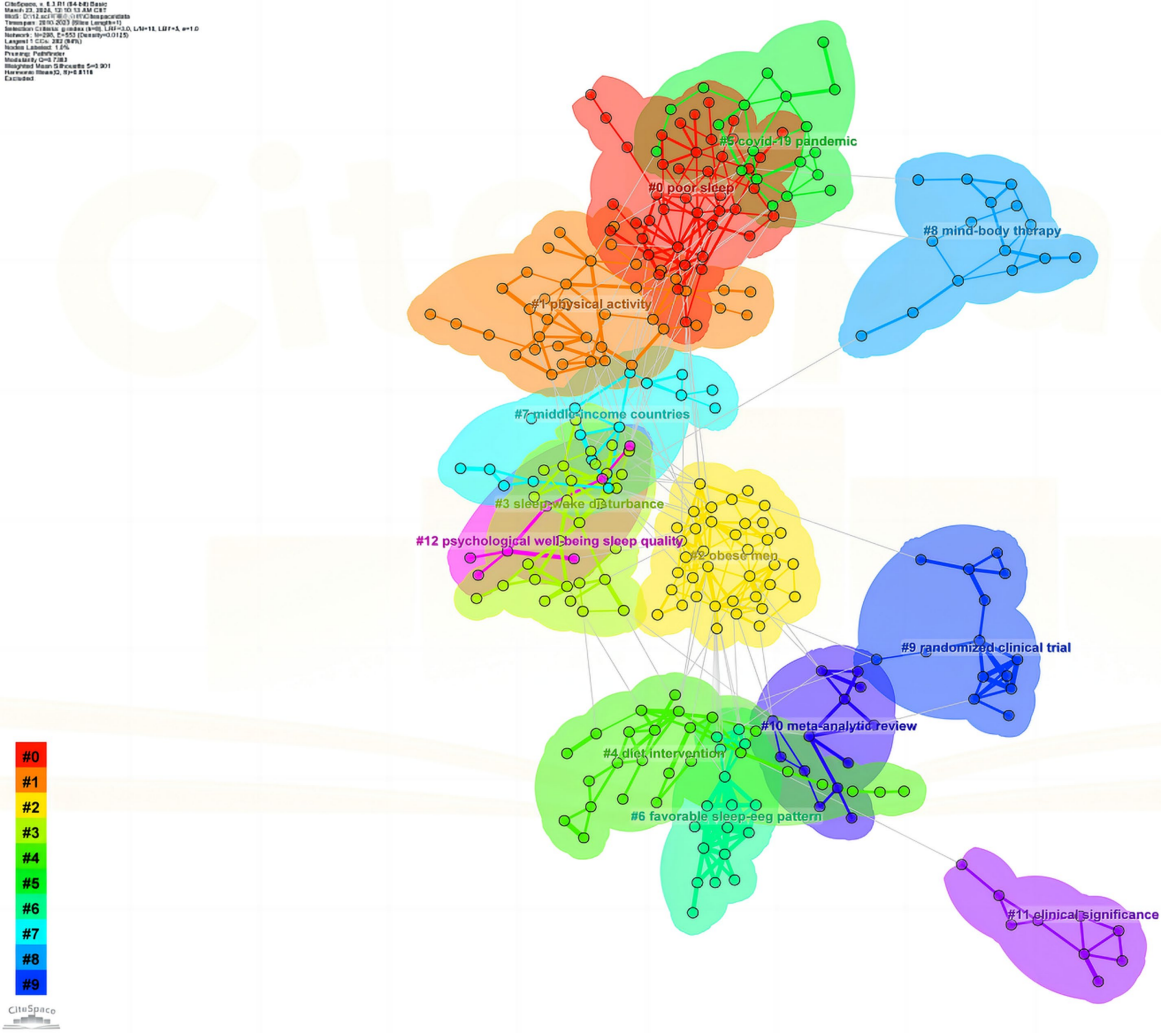
Clustering of references.

By clustering references in the field of exercise therapy for insomnia, 12 clustered themes were obtained, as shown in Figure 5: #0 poor sleep, #1 physical activity, #2 obese men, #3 sleep–wake disturbance, #4 diet intervention, #5 Covid-19 pandemic, #6 favorable sleep-EEG pattern, #7 middle-income countries, #8 mind–body therapy, #9 randomized clinical trial, #10 meta-analytic review, #11 clinical significance, and #12 psychological well-being and sleep quality. These represent the most recent research directions in the field of exercise therapy for insomnia and can be themes for future research in this field.

### 3.7 Keywords

**Figure 6:**
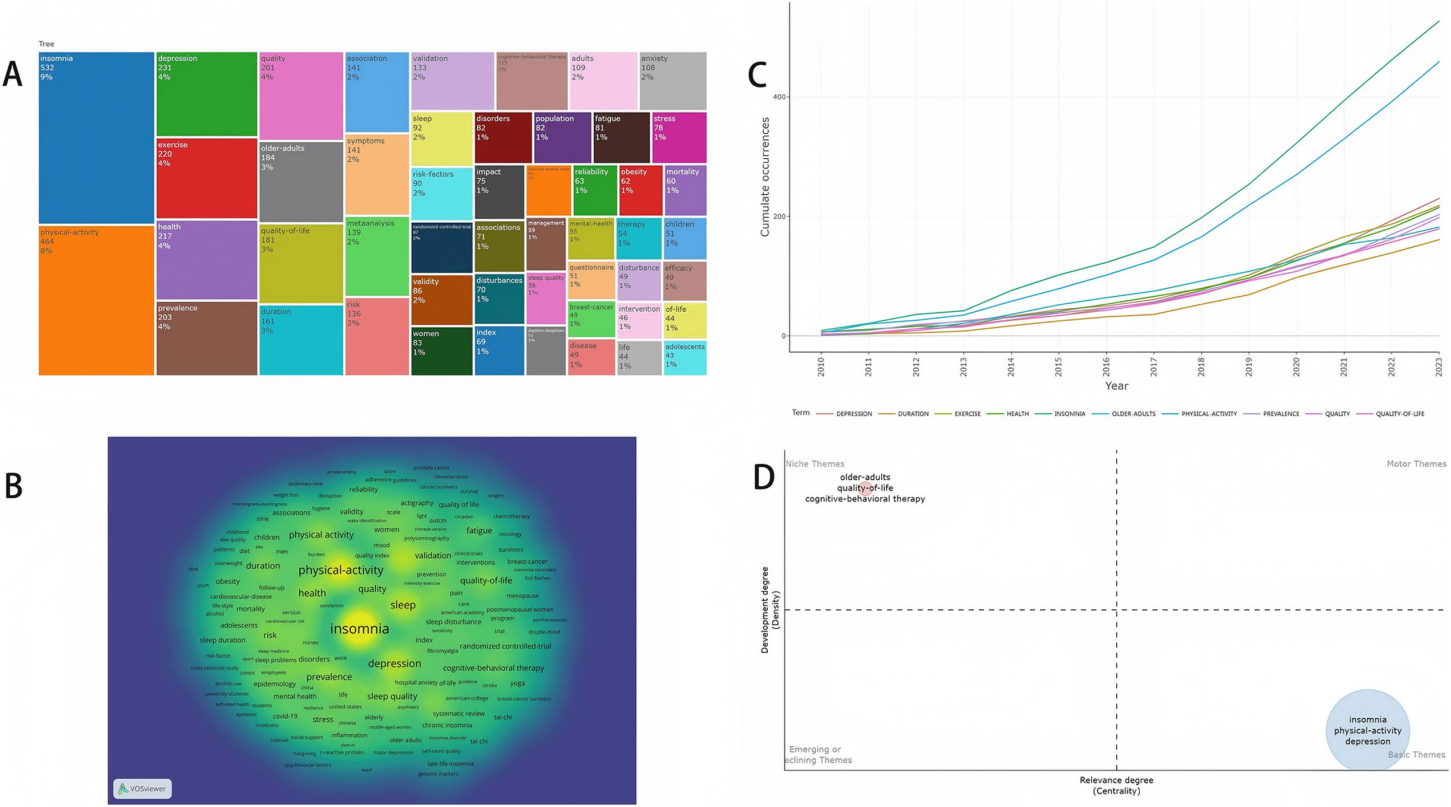
**(A)** Treemap of the research areas in exercise and insomnia. **(B)** Keyword density visualization. **(C)** Growth trend of the top 10 keywords. **(D)** Keyword thematic map.

Keyword analysis can help researchers find keywords used with high frequency in a research field as well as the correlation between different keywords to quickly identify the topic of the research field. There are approximately 4,501 keywords in the field of exercise for insomnia. Figure 6A shows that the most frequently occurring keywords in this field were insomnia (532 times) physical activity (464 times) depression (231 times) exercise (220 times) health (217 times) prevalence (203 times) quality (201 times) older adults (184 times) quality of life (181 times) and duration (161 times)

Figure 6B presents the keyword density visualization, indicating that the main keywords in this research area were insomnia, physical activity, and depression. Figure 6C shows that the frequency of the first 10 keywords in this research area increased over time, with insomnia and physical activity growing the fastest. Figure 6D shows the keyword topic terms, where the niche themes include older adults, quality of life, cognitive-behavioral therapy, and insomnia. The basic themes included insomnia, physical activity, and depression. By analyzing the above keywords, we can predict that the future keywords in the research field of exercise therapy for insomnia may be “insomnia,” “exercise,” “depression,” “older adults,” and “quality of life.”

## 4 Discussion

### 4.1 Essential information

This study analyzed 1,419 papers on exercise therapy for insomnia between 2010 and 2023. We used CiteSpace, VOSviewer, and the Bibliometrix package based on R language software to analyze the publications, countries and regions, institutions, journals, authors, references, keywords, number of articles issued, co-citation frequency, etc., to visualize and intuitively show the current status and hotspots of research in this field.

Publication analysis found that the number of publications in the field of exercise for insomnia research increased annually from 2016 to the present, and the number of publications in 2023 will reach a new all-time high with 230 articles. This indicates that an increasing number of researchers are interested in exercise therapy for insomnia and that more people are investing in this research field.

When analyzing countries and regions, we found that this research field is mainly concentrated in Western Europe, East Asia, and North America, with the United States and China having the highest numbers of publications. Similarly, we found that the institution with the highest number of publications in this field was the University of California System in the United States, and six of the top 10 institutions with the highest number of publications were from the United States.

Moreover, the journal with the most publications was the *International Journal of Environmental Research and Public Health*, a quarterly journal from Switzerland that focuses on medicine, public health, and environmental and occupational health. Markus Gerber from Switzerland has the highest number of publications in this field of research, focusing on psychology, psychiatry, neuroscience, neurology, and exercise science. Four of the top 10 authors were from Switzerland, indicating that Switzerland has some of the most cutting-edge researchers in the field of exercise therapy for insomnia. “The Effects of Physical Activity on Sleep: A Meta-Analytic Review” by Kredlow MA et al. (16) is the most cited reference in this area of research. This article analyzed 63 papers that included 66 items meeting the criteria and thus provided support for exercise as an evidence-based intervention to improve perceived and objective sleep metrics in healthy individuals.

In conclusion, although the number of publications in the research field of exercise therapy for insomnia is increasing, there is an international research imbalance. This field should be strengthened through international exchanges and national cooperative research.

### 4.2 Research trends and hotspots

Keywords form the core overview of a paper and keyword analysis can quickly capture research hotspots in a subject area. This study conducted an analysis of keyword emergence clustering and development trends in the research on exercise therapy for insomnia based on the R language through the Bibliometrix package. Accordingly it identified the following research trends and hotspots in this research area: “insomnia,” “exercise,” “depression,” “older adults,” and “quality of life.” By analyzing these keywords in detail the current development of the research field of exercise for insomnia is further dissected

#### 4.2.1 Insomnia

Insomnia is a common neurological disorder categorized as short-term, chronic, or other (18). Insomnia may cause harmful effects such as memory loss, premature aging, and decreased immunity, and severe long-term insomnia may cause other complications such as mental disorders and hypertension (19–21). In the pathogenesis of insomnia, certain nuclei of the brain, neurotransmitters, transmitter systems, growth factors, hormones, and other substances act on the sleep center of the human body under the influence of the environment, psychology, disease, drugs, and genetics; this results in abnormalities in the central neurotransmitter system, which lead to insomnia (1, 22, 23).

As the study of insomnia continues to deepen, it has shifted from symptomatology to etiology (19). Studies have shown that chronic inflammation, stressful events, and emotional trauma can alter the hypothalamic–pituitary–adrenal axis, leading to an increase in the secretion of cortisol and thyrotropin-releasing hormones in the body, which can act on the nervous system to change the state of sleep or mood and induce insomnia (24, 25). A study on the pathogenesis and risk factors of insomnia found that the risk of insomnia was significantly higher in women than in men (26). This risk is also higher in people with physical illnesses such as heart disease and stroke, and mental illnesses such as depression (27, 28).

#### 4.2.2 Exercise

Exercise is an effective treatment for insomnia symptoms (29, 30). Current research on exercise for the treatment of insomnia has focused on analyzing exercise’s mechanisms, types, and duration. Early studies showed that aerobic exercise improves sleep quality in older adults with chronic insomnia and indicated a positive relationship between improvement in sleep quality and improvement in maximum heart rate in the exercise group. The effect of exercise on sleep quality may be mediated by mechanisms other than cardiorespiratory fitness (31). Passos et al. reported that exercise affects the immune system by decreasing CD4 and CD8 cell counts, thereby improving sleep quality in patients with insomnia (29). In a randomized controlled trial, tai chi exercise reduced cellular inflammatory responses and decreased the expression of genes encoding pro-inflammatory mediators in patients with insomnia. This study revealed the potentially beneficial effects of exercise on insomnia and inflammation (32). In addition, the current mechanistic analysis of exercise for insomnia includes the analysis of neurotransmitters, growth factors, and psychological factors (33–35).

Many studies have shown that aerobic exercise has the most significant effect on patients (36, 37). Because the improvement in sleep by aerobic exercise is continuous and moderate, overexercise may lead to over-fatigue of the body and affect sleep. However, tai chi and yoga, as physical and mental exercises, have been more frequently used as interventions for the treatment of insomnia (38–41). The effects of aerobic exercise, tai chi, and yoga on insomnia need to be explored to determine the intervention mechanism. These forms of exercises are future research directions in this field.

#### 4.2.3 Depression

There is a strong correlation between depression and insomnia (42). Insomnia may be a symptom of depression, and chronic insomnia may lead to a depressive mood. Symptoms of depression, a common mental disorder, include insomnia, early awakening, and lethargy, and insomnia may be one of these symptoms, especially in the early stages of depression (43). Insomnia can lead to physical and psychological discomfort that can affect mood. Chronic insomnia may lead to low mood, anxiety, and irritability, which may lead to depression (44).

In a randomized controlled trial, exercise training reduced depressive symptoms in patients with chronic primary insomnia (11). One study reported that depression is a mediator of insomnia and that control of depression after exercise can be achieved by utilizing neurotransmitters, which in turn reduce insomnia (10). Multiple biological mechanisms are involved in the treatment of depression using exercise, including neuroplastic changes, immune system effects, oxidative stress, and inflammation, all of which work together to make exercise an effective antidepressant.

#### 4.2.4 Older adults

Insomnia is a common problem among older adults, and according to statistics, the prevalence of insomnia among people aged over 65 years is 20–50% (45). As age increases, physiological systems gradually age, and the sleep structure of older adults changes. Many older adults experience a significant reduction in sleep duration, and some may even experience severe insomnia. Previous studies have indicated that aerobic exercise significantly affects mood and sleep quality in older adults with insomnia (11). In recent years, studies have also noted that walking or tai chi is a more suitable alternative to complementary therapies for older adults with insomnia (46).

#### 4.2.5 Quality of life

The Quality-of-Life Scale is an instrument used to assess an individual’s quality of life, including physical functioning, psychological status, social functioning, material well-being, and feelings of health. In studies on exercise therapy for insomnia, quality of life is an indicator of patient rehabilitation assessment. Quality of life enables the assessment of a patient’s state of health and effectiveness of treatment.

### 4.3 Limitations

This is the first bibliometric analysis of the research area of exercise therapy for insomnia. Although we conducted a rigorous analysis of this research area, the study may have issues due to research design or methodology limitations. Despite our best efforts to control for these limitations, they may have impacted our findings and conclusions. First, database and language limitations may have led to a lack of awareness of exercise therapy’s comprehensiveness and globalization trends for insomnia, especially when research results from some non-English-speaking countries were not included. Second, keyword limitations may have caused us to miss some potentially important research directions in identifying research hotspots, thus affecting our prediction of future developments in the field. Therefore, although this paper provides an important perspective for bibliometric analysis in the field of exercise for insomnia research, we suggest that future studies could expand the literature search to include more databases and languages in order to validate our conclusions further and to conduct in-depth comparative analyses of studies in different language contexts. In addition, researchers should adopt more comprehensive keyword analysis methods combined with automated text mining techniques to reduce human bias and improve the accuracy and comprehensiveness of hotspot identification.

## 5 Conclusion

This study analyzed the research status and hotspots of research on exercise therapy for insomnia conducted during 2010–2023 using bibliometric methods. It visually presented the key information in the field, which can provide researchers clear and intuitive references. This study found that the number of publications in the research field of exercise therapy for insomnia has been increasing, and this field has received more attention. The United States and China hold important positions in this research field, whereas Switzerland has the highest talent. The hotspots and trends in this field of research identified through keyword analysis are “insomnia,” “exercise,” “depression,” “older adults,” and “quality of life.”

## Data Availability

Publicly available datasets were analyzed in this study. The data that support the findings of this study are available from the corresponding author upon reasonable request.
